# Correction: Improving the inhibitory effect of CXCR4 peptide antagonist in tumor metastasis with an acetylated PAMAM dendrimer

**DOI:** 10.1039/c8ra90102c

**Published:** 2018-12-18

**Authors:** Changliang Liu, Hongyang Duan, Zijian Zhao, Wenzhe Li, Lilusi Ma, Xiaocui Fang, Chen Wang, Yanlian Yang

**Affiliations:** CAS Key Laboratory of Standardization and Measurement for Nanotechnology, CAS Key Laboratory of Biological Effects of Nanomaterials and Nanosafety, CAS Center for Excellence in Nanoscience, National Center for Nanoscience and Technology, and University of Chinese Academy of Sciences Beijing 100190 P. R. China Beijing 100049 P. R. China fangxc@nanoctr.cn wangch@nanoctr.cn yangyl@nanoctr.cn +86-10-82545561 +86-10-82545559; Academy for Advanced Interdisciplinary Studies, Peking University Beijing 100871 China

## Abstract

Correction for ‘Improving the inhibitory effect of CXCR4 peptide antagonist in tumor metastasis with an acetylated PAMAM dendrimer’ by Changliang Liu *et al.*, *RSC Adv.*, 2018, **8**, 39948–39956.

The version of affiliation *a* was incomplete in the published article, while the correct version is shown here.

The Royal Society of Chemistry apologises for these errors and any consequent inconvenience to authors and readers.

## Supplementary Material

